# Editorial

**Published:** 1902-10-15

**Authors:** 


### EDITORIAL.

We recently read the report of a long discussion of a paper on the use of cocain for dental purposes, in which every speaker confessed that he made very little use of it in his practice, he was afraid of it and never felt sure what complications would arise. There are people who discuss the use of tea, coffee or whisky on much the same kind of evidence. They don't know much about these drugs because they have never become familiar with them. The habitual whisky drinker don't realize that there is a lethal dose of his favorite beverage until it gets the better of him in an unguarded moment. May it not be so in the use of cocain, a far greater boon to humanity than whisky, and should we not become sufficiently familiar with it to be able to avail ourselves of its wonderfully helpful properties?

A paper was recently read, before one of our state societies, which contained the essentials of a most valuable contribution as to the use of a certain drug in dental practice. But either from the manifest ignorance or carelessness of the essayist—and perhaps we should also include the editor of the journal in which it appeared, it's value will undoubtedly be lost, for a time at least. There are lots of good workers in our ranks who are daily working out valuable details of practice, but who unfortunately think so little of their professional responsibilities that they will not take time to communicate their ideas to the profession, except when urgently requested to do so by some dental society officer who forces them to an unwilling task, which they defer to the last day in the evening before starting to the meeting. Consequently they neither entertain nor instruct, and draw small credit. Let's do better!

---

A CONSIDERABLE controversy of local as well as national interest has been precipitated by the request of the New York State Dental Society to the Board of Regents of New York State, that it confer the D.D.S. degree on all dentists who have previously had the M.D.S. degree conferred upon them by the New York State Dental Society, which now does not possess this power. This degree has been conferred upon about one hundred and twenty-five dentists, many of whom already have the D.D.S. degree, some are dead and quite a few do not now practice in the State or at all. So that it is probably true that not over seventy-five persons are interested. But of these some are strongly of the opinion that they are entitled to the degree of D.D.S. and are doing all they can to have this action taken. We do not believe much practical injury will be done if this effort is successful, so far as lowering the standard of the D.D.S. degree which the Colleges have so strenously contended during the past fifteen years should be conferred only on evidence of actual attendance on the course of lectures, as prescribed from time to time by the National Association of Dental Faculties. As those who now hold the M.D.S. possess the equivalents or better in many cases than the curriculum calls for. It however establishes a precedent which may give trouble in the future and is in direct conflict with the present conditions which are so well known everywhere that no one now thinks of asking for an honorary degree from a dental college. We do not see the necessity for any such action, as the change in degree would not benefit those who now have the degree in any way. The M.D.S. degree has not, so far as we know been discredited officially anywhere, and it was so wisely conferred that no personal odium attaches to it. It is much easier to tear down than to construct, and we fear the influence of such action would be to undermine the work that has been so well and laboriously accomplished by the concerted efforts of our educational institutions. We therefore hope no action may be taken.

